# MicroRNA expression in ovarian carcinoma and its correlation with clinicopathological features

**DOI:** 10.1186/1477-7819-10-174

**Published:** 2012-08-27

**Authors:** Heejeong Lee, Chul Soo Park, Georgios Deftereos, Janice Morihara, Joshua E Stern, Stephen E Hawes, Elizabeth Swisher, Nancy B Kiviat, Qinghua Feng

**Affiliations:** 1 Department of Pathology, College of Medicine, The Catholic University of Korea, 327, Sosa-ro, Wonmi-gu, Bucheon, Gyeonggi-do, 420-717, South Korea; 2 Department of Internal Medicine, College of Medicine, The Catholic University of Korea, 62 Yoido-dong, Youngdeungpo-gu, Seoul, 150-713, South Korea; 3 Department of Pathology, Allegheny General Hospital, 320 East North Avenue, Pittsburgh, PA, 15212, USA; 4 Department of Pathology, School of Medicine, University of Washington, 1959 North East Pacific Street, Seattle, WA, 98195, USA; 5 Department of Epidemiology, School of Public Health, University of Washington, 1959 North East Pacific Street, Seattle, WA, 98195, USA; 6 Department of Obstetrics and Gynecology, School of Medicine, University of Washington, 1959 North East Pacific Street, Seattle, WA, 98195, USA

**Keywords:** miRNA, Ovarian tumor, Her2/neu, Survival

## Abstract

**Background:**

MicroRNA (miRNA) expression is known to be deregulated in ovarian carcinomas. However, limited data is available about the miRNA expression pattern for the benign or borderline ovarian tumors as well as differential miRNA expression pattern associated with histological types, grades or clinical stages in ovarian carcinomas. We defined patterns of microRNA expression in tissues from normal, benign, borderline, and malignant ovarian tumors and explored the relationship between frequently deregulated miRNAs and clinicopathologic findings, response to therapy, survival, and association with Her-2/neu status in ovarian carcinomas.

**Methods:**

We measured the expression of nine miRNAs (miR-181d, miR-30a-3p, miR-30c, miR-30d, miR-30e-3p, miR-368, miR-370, miR-493-5p, miR-532-5p) in 171 formalin-fixed, paraffin-embedded ovarian tissue blocks as well as six normal human ovarian surface epithelial (HOSE) cell lines using Taqman-based real-time PCR assays. Her-2/neu overexpression was assessed in ovarian carcinomas (n = 109 cases) by immunohistochemistry analysis.

**Results:**

Expression of four miRNAs (miR-30c, miR-30d, miR-30e-3p, miR-370) was significantly different between carcinomas and benign ovarian tissues as well as between carcinoma and borderline tissues. An additional three miRNAs (miR-181d, miR-30a-3p, miR-532-5p) were significantly different between borderline and carcinoma tissues. Expression of miR-532-5p was significantly lower in borderline than in benign tissues. Among ovarian carcinomas, expression of four miRNAs (miR-30a-3p, miR-30c, miR-30d, miR-30e-3p) was lowest in mucinous and highest in clear cell samples. Expression of miR-30a-3p was higher in well-differentiated compared to poorly differentiated tumors (*P* = 0.02), and expression of miR-370 was higher in stage I/II compared to stage III/IV samples (*P* = 0.03). In multivariate analyses, higher expression of miR-181d, miR-30c, miR-30d, and miR-30e-3p was associated with significantly better disease-free or overall survival. Finally, lower expression of miR-30c, miR-30d, miR-30e-3p and miR-532-5p was significantly associated with overexpression of Her-2/neu.

**Conclusions:**

Aberrant expression of miRNAs is common in ovarian tumor suggesting involvement of miRNA in ovarian tumorigenesis. They are associated with histology, clinical stage, survival and oncogene expression in ovarian carcinoma.

## Background

Ovarian carcinoma is the most lethal form of gynecological malignancy in Korea, with a burden of 4,536 new cases and the highest mortality/incidence (M/I) ratio (44.8%) among the female genital tract cancers compared to uterine cervix (26.0%) or corpus (12.8%) cancers from 2003 to 2005 [1]. The majority of cases are diagnosed in late stages when metastases are already present and survival is poor [2]. Current screening assays, such as serum CA125 and transvaginal ultrasound, lack both the sensitivity and specificity needed for effective early detection of ovarian carcinoma. Platinum-based chemotherapy is the standard treatment for patients with advanced ovarian carcinoma, and current prognostic markers include International Federation of Gynecology and Obstetrics (FIGO) stages, volume of residual tumor after cytoreductive surgery, patient age, histopathological grade, and DNA ploidy [3]. A better understanding of molecular alterations in ovarian carcinoma is necessary to identify novel targets for early detection and improved treatment.

MicroRNAs (miRNAs) are small noncoding RNAs that have been implicated in tumor development. They regulate target gene expression either by mRNA degradation or by translation repression [4-11]. Each miRNA may regulate up to hundreds of target genes [11]. During tumor development, aberrant expression of miRNAs can either inactivate tumor suppressor genes or activate oncogenes, thus promoting tumor formation [4-6,8-10,12]. Because expression of miRNAs is tissue-specific, detectable in blood [13], and correlates with clinical cancer behaviors [14], miRNAs are potential valuable biomarkers.

Numerous studies have identified both upregulated and downregulated miRNAs in ovarian carcinoma samples, using both candidate gene [6,13,15] and global profiling [16-25] approaches. Aberrant expression of miRNAs has also been associated with tumor histology [6,19], response to therapy [21,24] and survival [26]. However, most studies have compared miRNA expression to either normal ovarian tissues or human ovarian surface epithelial (HOSE) cell lines; it is unknown whether these samples represent miRNA expression in normal ovarian epithelial cells [18,27].

We previously identified methylation of three ovarian carcinoma-specific genes (MINT31, RASSF1, and CDH13) significantly associated with Her-2/neu overexpression [28]. Using the TargetScan database (http://www.targetscan.org) we identified nine miRNAs that regulate these three genes: miR-368 targeting MINT31, miR-181d, miR-30a-3p, miR-30c, miR-30d, miR-30e-3p, miR-370, miR-493-5p and miR-532-5p targeting CDH13, and miR-181d targeting RASSF1. In the present study, we determined the expression of these nine miRNAs in benign, borderline and ovarian carcinoma tissues, as well as HOSE cell lines and normal ovarian tissues by quantitative real-time reverse transcription-PCR. In addition, miRNA expression was correlated with Her-2/neu expression as well as clinicopathologic status in ovarian carcinoma.

## Methods

### Clinical ovarian tissue samples

A total of 171 archived formalin-fixed, paraffin-embedded (FFPE) tissue blocks of normal ovaries (22), and benign (17), borderline (23) or ovarian carcinoma (109) were obtained from the pathology department of Bucheon St. Mary’s Hospital, located in the Gyeongin region of South Korea, from 1994 to 2004. The patients’ ages ranged from 16 to 88 years, with a median of 52 years. Median ages of normal, benign, borderline and carcinoma patients were 55 years (range 49 to 68 years), 43 years (range 16 to 88 years), 35 years (range 17 to 72 years), and 52 years (range 20 to 85 years) respectively, with patients with normal pathology significantly older than women with benign (*P* = 0.02) or borderline (*P* = 0.0006) neoplasms, and women with carcinomas significantly older than women with borderline (*P* = 0.0007) neoplasms. None of the study subjects had a known family history of breast and/or ovarian cancer, or BRCA1 germ-line mutations. Histologic diagnoses were reviewed by two independent pathologists. Normal and benign tissues were obtained from patients who had oophorectomy for benign uterine pathologies. For carcinoma tissue blocks, only primary tumor blocks before treatment and those with more than 80% tumor content were used for this study. Detailed pathological features of benign, borderline, and carcinomas are presented in Table 1.

**Table 1:** Pathological features of 149 ovarian samples

**Tissue diagnosis**	**Number (%)**
**Benign tumors**	**17 (11.4)**
Serous	7
Mucinous	10
**Borderline tumors**	**23 (15.4)**
Serous	8
Mucinous	15
**Carcinomas**	**109 (73.2)**
**Histology**	
Serous	66
Mucinous	24
Endometrioid	12
Clear cell	7
Grade and differentiation*	
Well-differentiated (grade 1)	19
Moderately differentiated (grade 2)	57
Poorly differentiated (grade 3)	32
FIGO stage*	
I	36
II	8
III	58
IV	6

*Histologic grade and International Federation of Gynecology and Obstetrics (FIGO) stage missing for one ovarian carcinoma case.

Of the 109 patients with ovarian carcinoma, 41 patients were lost due to transferring to a different hospital, the remaining 68 patients were followed after surgery, both treatment and survival information was extracted from their medical records. Of these patients, 62 received platinum-based chemotherapy and 32 of them subsequently received paclitaxel chemotherapy, the remaining 6 patients did not have any chemotherapy. Among patients who received chemotherapy, platinum-sensitive cases were defined as those who had a progression-free survival of at least six months after the completion of chemotherapy. Platinum-resistant cases were defined as patients who progressed through, had persistent disease at the completion of chemotherapy, or had recurrent disease within six months after the chemotherapy. Overall survival was calculated from the initiation date of primary chemotherapy until the death or the last follow-up visit (up to 122 months). Disease-free survival was defined as the interval from the initiation date of primary chemotherapy to the date of progression.

### HOSE cell lines

Six normal HOSE cell lines were established from fresh ovarian scrapings of patients with benign ovarian conditions at the time of surgery and were subsequently immortalized with HPV16 E6/E7 oncogenes. These cell lines were cultured in mammary epithelial cell growth medium (MEGM) (BulletKit, Clonetics; Lonza Group Ltd, Basel, Switzerland) supplemented with 1% fetal bovine serum, 1% penicillin G sodium and streptomycin sulfate. Epithelial purity was confirmed by cytokeratin expression using immunohistochemistry analysis.

### RNA extraction

Total RNA from FFPE tissues was extracted using RecoverAll^TM^ Total Nucleic Acid Extraction Kit following the manufacturer’s protocol (Ambion Inc., Austin, TX, USA). RNA was extracted from HOSE cell lines using the same kit simply by omitting the deparaffination steps. RNA concentration and purity was assessed using UV spectrophotometer.

### Reverse transcription and quantitative real-time PCR (qPCR)

Reverse transcription and quantitative polymerase chain reactions were performed for the following miRNAs: miR-181d, miR-30a-3p, miR-30c, miR-30d, miR-30e-3p, miR-368, miR-370, miR-493-5p, and miR-532-5p using the TaqMan^TM^ MicroRNA Reverse Transcription Kit and the TaqMan^TM^ MicroRNA Assays in triplicate (Applied Biosystems, Foster City, CA, USA). U6 small nuclear 2 (RNU6b) was used to normalize input of total small RNA. Absolute quantification for each miRNA as well as RNU6b was performed using a standard curve generated by serial dilution of reverse transcribed total RNA extracted from VK2 cells, and expression of each miRNA was presented as the ratio between miRNA and RNU6b (RQ).

### Immunohistochemical analysis of Her-2/neu overexpression

Archived ovarian carcinoma tissue blocks were used for immunohistochemistry analysis of Her2/neu overexpression. Four-micron sections of FFPE tissue were cut and placed on Superfrost Plus microscope slides (VWR International, Radnor, PA, USA). The tissue sections were deparaffinized and rehydrated through graded alcohols. Endogenous peroxidase activity was blocked by incubation in 3% H_2_O_2_. Antigen retrieval was carried out with 0.01 M citrate buffer pH 6.0 and microwave heat induction. Approximately 100 μl of Her2/neu primary rabbit polyclonal antibody (diluted 1:8,000) (DakoCytomation, Glostrup, Denmark) or antibody diluents lacking the primary antibody was applied to each slide. The slides were washed, and biotinylated anti-rabbit antibody (diluted 1:500) (Vector Laboratories Burlingame, CA, USA) was applied. After a second wash, the avidin-biotin-peroxidase complex (Vector Laboratories) was applied. Color development was accomplished by incubation in diaminobenzidine with 3% H_2_O_2_ as a substrate, and nickel chloride enhancement. The slides were counterstained with methyl green, dehydrated through graded alcohols, cleared in xylene, and coverslipped with permanent mounting media. All cases were reviewed and scored without knowledge of other laboratory or clinical results. A case was scored as positive for Her-2/neu overexpression if it exhibited a staining intensity of 2+ to 3+ with circumscribed membrane staining in more than 10% neoplastic cells, but not in normal cells. A case was scored as negative for Her-2/neu overexpression if staining was absent (score 0), staining intensity was 1+ or less, or if circumscribed membrane staining was absent regardless of staining intensity. Each batch of slides was run with a known tissue lacking Her-2/neu overexpression as the negative control and a known tissue with Her-2/neu overexpression as the positive control, as well as control breast cancer cell lines with known levels of Her2/neu expression (MDA-231 (0), MDA-175 (1+) and SK-BR-3 (3+)) (HercepTest™ Interpretation Manual, DakoCytomation) [29].

### Statistical analysis

Expression values of all nine microRNAs were log_10_ transformed in order to normalize the distribution of these measurements for all statistical analyses. ANOVA was used to compare patients’ age across histology and clinicopathological findings with respect to the RQ of miRNA expression. In the final analysis, multivariable adjustments were made to adjust for the potentially confounding effects of histological type, stage, grade, and Her2/neu expression. All *P*-values were adjusted for multiple comparisons using false discovery rates. Cox proportional hazards models were used to assess the associations between miRNA expression and overall and disease-free survival. From these models, risk estimates, expressed as hazard ratios (HR), and 95% confidence intervals (CI) were calculated. A two-sided 0.05 test level determined statistical significance for all analyses. All analyses were conducted using SAS version 9.2 (SAS Institute Inc., Cary, NC, USA).

Informed consent was obtained according to procedures approved by the Human Subjects Committee of the Catholic University of Korea and University of Washington.

## Results

### MicroRNA expression in clinical samples

**Figure 1:**
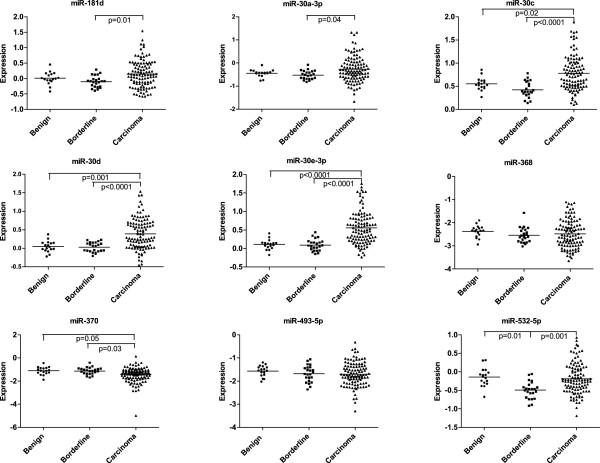
**miRNA expression in benign, borderline tumors and ovarian carcinomas.** Expression of each miRNA in benign, borderline tumors and ovarian carcinomas was shown by an individual scatter plot. All significant comparisons were labeled (*P* <0.05).

We detected significant difference of miRNAs expression among HOSE cell lines, normal ovaries and benign ovarian neoplasms (see Additional file 1). Because of the potential artifacts introduced by *in vitro* culturing and transformation in HOSE cell lines and the high content of stroma cells in normal ovarian tissues, we decided to use benign ovarian neoplasms as normal control. Expression of four miRNAs (miR-30c, 30d, 30e-3p, and 370) was significantly different between benign neoplasms and ovarian carcinoma: expression of miR-30c (*P* = 0.02), miR-30d (*P* = 0.001) and miR-30e-3p (*P* <0.0001) was higher while expression of miR-370 (*P* = 0.05) was lower in ovarian carcinomas. In addition, expression of miR-532-5p (*P* = 0.01) was lower in borderline than in benign neoplasms. Finally, we also observed significant differences in expression between borderline neoplasms and carcinomas for seven out of nine miRNAs (Figure 1).

### Association of microRNA expression and histological types in ovarian carcinomas

**Figure 2:**
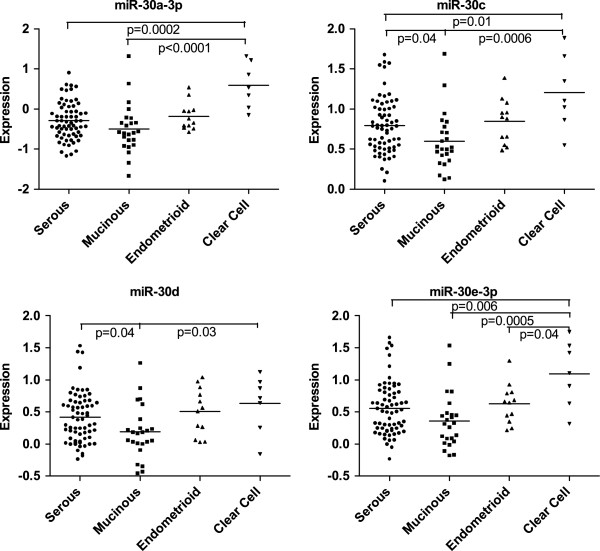
**Differential miRNA expression by histological types in ovarian carcinomas.** Scatter plots of expression of miR-30a-3p, miR-30c, miR-30d and miR-30e-3p indicated significant differences among different histological types.

Among ovarian carcinoma, we determined whether expression of specific miRNAs was associated with histological types (Figure 2). Expression of miR-30a-3p (*P* = 0.0002 for serous and *P* < .0001 for mucinous), miR-30c (*P* = 0.01 for serous and *P* = 0.0006 for mucinous), and miR-30e-3p (*P* = 0.006 for serous and *P* = 0.0005 for mucinous) were significantly higher in clear cell samples than in both serous and mucinous samples. Expression of miR-30d (*P* = 0.03) was significantly higher in clear cell samples than in mucinous samples. Lastly, expression of miR-30c (*P* = 0.04) and miR-30d (*P* = 0.04) were significantly higher in serous carcinomas than in mucinous samples, and expression of miR-30e-3p (*P* = 0.04) was significantly higher in clear cell than in endometrioid carcinomas.

### Association of microRNA expression and Her2/neu status, stage and grade in ovarian carcinomas

In order to assess the independent associations between miRNA expression and histological type, stage, grade and Her-2/neu overexpression in ovarian carcinomas, we conducted multiple ANOVA. Expression of miR-30a-3p was significantly higher in well-differentiated carcinomas (grade 1) compared to poorly differentiated carcinomas (grade 3) (*P* = 0.02) and expression of miR-181d was marginally higher in well-differentiated carcinomas (grade 1) compared to poorly differentiated carcinomas (grade 3) (*P* = 0.08). In addition, expression of miR-370 was significantly higher in stage I/II carcinomas compared to stage III/IV (*P* = 0.03) carcinomas.

**Figure 3:**
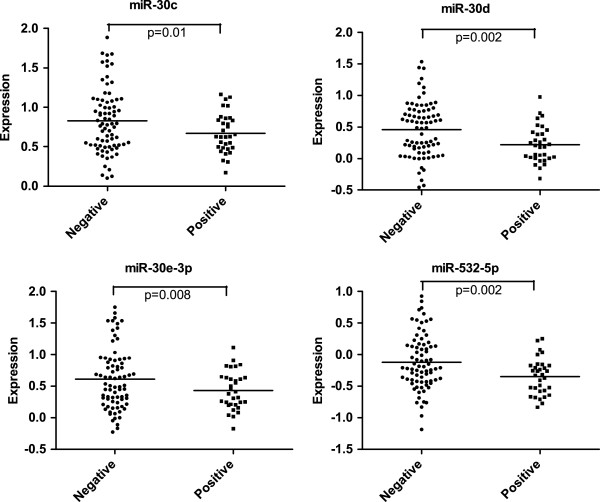
**Correlation of miRNA and Her2/neu expression in ovarian carcinomas.** Expression of miR-30c, miR-30d, miR-30e-3p and miR-532-5p was significantly downregulated among Her2/neu-positive ovarian carcinomas.

Of 109 carcinomas analyzed for Her2/neu overexpression, 38 (34.9%) were negative for Her2/neu expression, 39 (35.8%) had 1+ staining, 20 carcinomas (18.4%) had 2+ staining, and 12 (11.0%) had 3+ staining. We defined 2+ or 3+ staining cases as positive for Her2/neu expression in ovarian carcinoma (28). Expression of miR-30c (*P* = 0.01), miR-30d (*P* = 0.002), miR-30e-3p (*P* = 0.008) and miR-532-5p (*P* = 0.002) were significantly downregulated in Her2/neu-positive ovarian carcinomas (Figure 3). In addition, expressions of miR-181d (*P* = 0.07), miR-30a-3p (*P* = 0.12), miR-493-5p (*P* = 0.06) and miR-368 (*P* = 0.08) were downregulated in Her2/neu-positive ovarian carcinomas (data not shown).

### Association of microRNA expression and platinum sensitivity, disease recurrence and survival

Although expression of miR-30d was marginally upregulated in ovarian carcinomas which were platinum sensitive (*P* = 0.054) as opposed to those that were platinum resistant, none of the nine miRNAs were significantly associated with platinum sensitivity (data not shown). Interestingly, miR-30d was also downregulated in subjects who had recurrent disease (*P* = 0.15).

We investigated whether there was an association between miRNA expression overall and disease-free survival among carcinomas (Table 2). Among the 68 women available for overall survival analysis, 21 of them died during follow-up and their median survival time was 72 months. There were 58 women available for disease-free survival analysis, 30 of them recurred during follow-up and their median disease-free survival time was 32 months. All miRNAs except for three (miR-368, miR-370, and miR-493-5p) had hazard ratios less than one for every one unit increase in log_10_ miRNA expression for both overall and disease-free survival. In univariate models investigating overall survival, there was a moderate decreased risk of death associated with increased expression of miR-30d (*P* = 0.06). This association achieved significance after adjusting for age and stage (*P* = 0.02). In a univariate model investigating disease-free survival, there was a moderate decreased risk of recurrence for miR-181d (*P* = 0.25), miR-30c (*P* = 0.14), miR-30d (*P* = 0.07), and miR-30e-3p (*P* = 0.11). These associations became significantly associated with a decreased risk of recurrence after adjusting for age and stage.

**Table 2:** Cox proportional hazards regression analysis of the relation between miRNAs and overall and disease-free survival

	**Overall survival (n = 68)**				**Disease-free survival (n = 58)**			
	**Univariate analysis**		**Multivariate analysis**		**Univariate analysis**		**Multivariate analysis**	
	**HR (95% CI)**	***P*value**	**HR (95% CI)**	***P*value**	**HR (95% CI)**	***P*value**	**HR (95% CI)**	***P*value**
miR-181d	0.51 (0.19-1.41)	0.20	0.42 (0.14-1.23)	0.11	0.60 (0.26-1.41)	0.25	0.34 (0.12-0.94)	0.04
miR-30a-3p	0.50 (0.20-1.26)	0.14	0.48 (0.19-1.18)	0.11	0.56 (0.27-1.16)	0.12	0.46 (0.21-1.00)	0.05
miR-30c	0.48 (0.13-1.76)	0.27	0.37 (0.10-1.44)	0.15	0.44 (0.15-1.30)	0.14	0.27 (0.08-0.92)	0.04
miR-30d	0.33 (0.11-1.06)	0.06	0.25 (0.07-0.82)	0.02	0.41 (0.16-1.08)	0.07	0.33 (0.12-0.90)	0.03
miR-30e-3p	0.57 (0.20-1.61)	0.29	0.48 (0.17-1.37)	0.17	0.48 (0.20-1.18)	0.11	0.39 (0.16-0.94)	0.04
miR-368	1.60 (0.70-3.68)	0.27	1.79 (0.75-4.25)	0.19	0.84 (0.39-1.78)	0.64	0.92 (0.43-1.97)	0.84
miR-370	1.25 (0.65-2.39)	0.51	1.83 (0.78-4.31)	0.17	1.23 (0.74-2.06)	0.43	1.77 (0.93-3.34)	0.08
miR-493-5p	1.16 (0.47-2.84)	0.75	1.51 (0.57-4.00)	0.41	1.27 (0.58-2.77)	0.55	1.70 (0.74-3.94)	0.21
miR-532-5p	0.89 (0.32-2.52)	0.83	0.84 (0.30-2.36)	0.74	0.84 (0.34-2.07)	0.70	0.69 (0.27-1.74)	0.43

CI, confidence interval; HR, hazard ratio.

## Discussion

In the present study, we identified that expression of four miRNAs was significantly different between ovarian carcinomas and benign neoplasms, and differential miRNA expression was associated with histological types, grade and stage, as well as both disease-free survival and overall survival. Finally, miRNA expression was associated with Her2/neu oncogene expression. To the best of our knowledge, this is the first study that miRNA expression in ovarian carcinomas was compared to benign neoplasms by the quantitative real-time PCR method.

This study is the first to examine the relationship between miRNA alterations and Her-2/neu expressions in ovarian carcinomas. Several studies have identified specific miRNAs associated with Her2/neu expression in breast cancers. Using stepwise artificial neural networks (ANN) analysis, Lowery *et al*. identified a predictive miRNA signature (miR-520d, miR-181c, miR-302c, miR-376b, miR-30e) corresponding with Her2/neureceptor status [30]. Huang *et al*. [31] showed that miR-21 is upregulated in Her2/neu-positive breast cancer cells via the MAPK (ERK1/2) pathway. The expression of miR-221 and miR-222 was significantly elevated in Her2/neu-positive primary human breast cancer tissues when compared with Her2/neu-negative tissue samples [32].

We previously identified that methylation of three genes (MINT31, RASSF1, and CDH13) was significantly associated with Her2/neu overexpression in ovarian carcinoma from the United States [28]. In this study, we determined whether expression of miRNAs targeting these three genes was also associated with Her2/neu overexpression. Based on findings using the TargetScan software, miR-368 targets MINT31, miR-181d, 30a-3p, 30c, 30d, 30e-3p, 370, 493-5p, and 532-5p target CDH13, and miR-181d targets RASSF1. Interestingly, expression of miR-30c, 30d, 30e-3p and 532-5p were significantly higher in Her-2/neu-negative than in Her-2/neu-positive ovarian carcinoma. Based on our data, we hypothesize that CDH13 can be downregulated in ovarian carcinoma by two different pathways: in Her2/neu-negative ovarian carcinoma, CDH13-targeting miRNAs are sufficient to inactivate CDH13; in Her2/neu-positive ovarian carcinoma, these miRNAs are downregulated, CDH13 was inactivated through promoter DNA methylation. The high percentage of Her2/neu-positive ovarian carcinomas (29.4%) in the present study is likely due to the inclusion of 39% non-serous carcinomas (mucinous, endometrioid and clear cell types). The percentage of Her2/neu-positive serous carcinomas and non-serous carcinomas was 22.7% and 39.5% respectively in our study, which is consistent with what has been reported [33].

Our data on miRNA expression in ovarian carcinomas are consistent with what have been reported in the literature [6,19,26]. Wyman *et al*. showed that miR-30c, miR-30d and miR-30e were upregulated, while miR-493 was downregulated in ovarian carcinomas when compared to normal HOSE cell lines, and expression of miR-30a was specific to the clear cell histological type [19]. Zhang *et al*. reported downregulation of miR-368 and 370 in ovarian carcinomas [6]. Laios *et al*. showed significant downregulation of miR-30d in patients with recurrent ovarian carcinomas [26]. However, our study is unique in that we compared miRNA expression in ovarian carcinoma to benign or borderline tumor, whereas previous studies used HOSE cell lines or normal ovaries as controls. We identified that expression of four miRNAs (miR-30c, 30d, 30e-3p, and 370) was significantly different between ovarian carcinoma and benign tumor. Furthermore, expression of one miRNA was different between benign and borderline tumor and expression of seven miRNAs was different between borderline tumor and carcinoma. From our results, we hypothesize that these miRNAs are involved in ovarian tumorigenesis, especially at the stage of progression from borderline tumor to carcinoma. We identified that several miRNAs (miR-30a-3p, miR-30c, miR-30d, and miR-30e-3p) differently expressed in ovarian carcinoma with different histological types and this finding is pertinent to the fact that different histological types are biologically and pathogenetically distinct entities [16]. Although we did not detect downregulation of miR-368 in poorly differentiated carcinoma, which had been previously reported [6], we identified that high expression levels of miR-30a-3p and 370 were associated with well-differentiated tumor grade and early clinical stage in ovarian carcinoma, respectively.

Several studies reported association of expression of miR-30c and 370 with response to chemotherapy in ovarian carcinoma [21,24]. However, we did not detect any significant association between miRNA expression and platinum sensitivity in our study, although miR-30d were marginally upregulated in subjects who were platinum sensitive. The miRNA expression discrepancy we observed might be due to differences in type of specimens (cell lines versus tumor tissue), the inclusion of different histological subtypes, heterogeneity of the tumor, and RNA isolation protocols and detection platforms [27]. For example, more than 40% (44/108) of our carcinoma samples was early stage (I/II) and 39% of the early stage cases (17/44) were mucinous histology.

We showed that expression of miR-30d was significantly associated with both overall survival and disease-free survival in the multivariate analysis. In addition, miR-181d, miR-30c and miR-30e-3p were also significantly associated with disease-free survival in the multivariate analysis. Several studies have investigated the role of miRNA expression as prognostic markers in ovarian carcinoma [20,21,27,34,35]. Hu *et al*. showed that high expression of miR-200a was associated with recurrence-free survival and overall survival in ovarian carcinoma using multivariate Cox proportional analysis [36]. Leskelä *et al*. showed that high expression of miR-429 was associated with both recurrence-free survival and overall survival in ovarian carcinomas [37]. Wurz *et al*. showed that a higher ratio of miR-221/222 was associated with better overall survival [38]. A recent study in stage I ovarian carcinomas indicated that high expression of miR-200c was associated with better progression-free and overall survival [39].

Expression of miR-30c, 30d and 30e-3p has also been associated with survival in other types of cancer. For example, downregulation of expression of miR-30c has been associated with shorter progression-free survival in clear cell renal carcinoma [40] and breast cancer [41], but better survival in malignant mesothelioma [42], and low plasma level of miR-30e-3p was associated with shorter disease-free survival in non-small cell lung cancer [43]. However, upregulation of miR-30d was correlated with shorter time to recurrence and reduced overall survival in melanoma [44] and non-small cell lung cancer [45].

We detected significant differences among HOSE cell lines, normal ovarian tissues and benign neoplasms (supplemental data), this is consistent with what we had previously reported for DNA methylation analysis [28]. Since most of existing miRNA analysis studies used either HOSE cell lines or normal ovaries as normal controls, the reported results and conclusions should be interpreted with caution.

## Conclusions

We identified several miRNAs being aberrantly expressed in ovarian carcinomas when compared to benign or borderline ovarian tumors, suggesting that these miRNAs are involved in ovarian tumorigenesis. In addition, expression of some miRNAs was associated with distinct histological type while expression of others was associated with grade and clinical stage. Finally, expression of some miRNAs was associated with survival and oncogene expression in ovarian carcinomas. Future studies are needed to validate our findings and further elucidate the mechanism of miRNA expression regulation during ovarian tumorigenesis.

## Abbreviations

CI, confidence interval; FFPE, formalin-fixed, paraffin-embedded; FIGO stage, International Federation of Gynecology and Obstetrics; HOSE cell lines, human ovarian surface epithelial; HR, hazard ratio; MEGM, mammary epithelial cell growth medium; M/I ratio, mortality/incidence ratio; miRNA, microRNA; qPCR, quantitative real-time PCR; RNU6b, U6 small nuclear 2; RQ, ratio between miRNA and RNU6b.

## Competing interests

The authors have no commercial or other associations that might pose a conflict of interest.

## Authors’ contributions

HL, CP, GD, and JM conducted the experiments and HL drafted the manuscript. JS analyzed the data and SH assisted in the manuscript preparation. ES provided HOSE cell lines and assisted during the experiments and in the manuscript preparation. NK and QF conceptualized, edited, and revised the manuscript. All authors have read and approved the final manuscript.

## Supplementary Material

Additional file 1 **miRNA expression in HOSE cell lines, normal ovaries, and benign ovarian tumors.** Expression of miR-181d, miR-368, and miR-370 was significantly different between cell lines and normal ovaries as well as between cell lines and benign tumors. In addition, expression of miR-30c and miR-30e-3p was significantly different between cell lines and normal ovaries, and expression of miR-30c was significantly different between normal ovaries and benign tumors. (PDF 49 kb) Click here for file
