# Supplementary figures and images for: MicroRNA expression in ovarian carcinoma and its correlation with clinicopathological features
Source: World J Surg Oncol. 2012 Aug 27;10:174. doi: 10.1186/1477-7819-10-174 (PMC3449188; doi:10.1186/1477-7819-10-174)

miR-181d

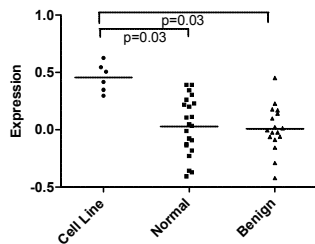

miR-30a-3p

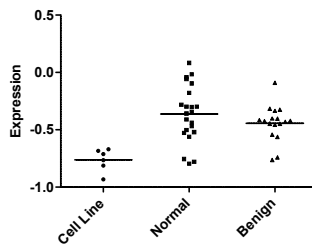

miR-30c

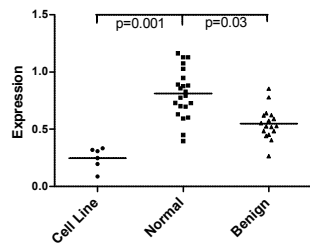

miR-30d

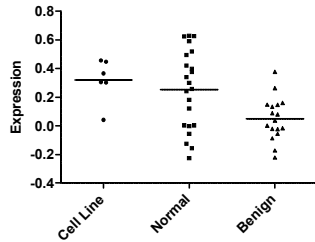

miR-30e-3p

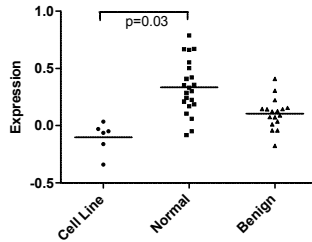

miR-368

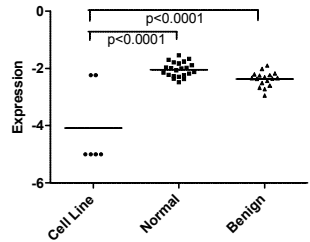

miR-370

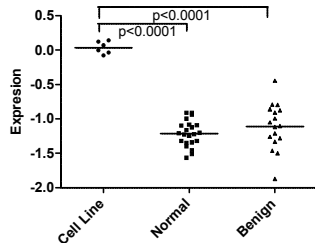

miR-493-5p

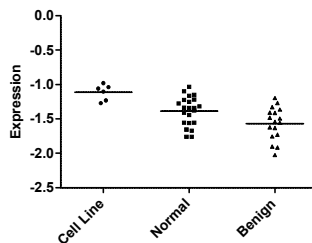

miR-532-5p

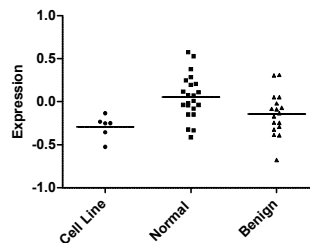

Supplement: Additional file 1 — miRNA expression in HOSE cell lines, normal ovaries, and benign ovarian tumors. Expression of miR-181d, miR-368, and miR-370 was significantly different between cell lines and normal ovaries as well as between cell lines and benign tumors. In addition, expression of miR-30c and miR-30e-3p was significantly different between cell lines and normal ovaries, and expression of miR-30c was significantly different between normal ovaries and benign tumors. (PDF 49 kb) [file 1477-7819-10-174-S1.pdf]
